# Supplementary material for: Hybrid Models and Biological Model Reduction with PyDSTool
Source: PLoS Comput Biol. 2012 Aug 9;8(8):e1002628. doi: 10.1371/journal.pcbi.1002628 (PMC3415397; doi:10.1371/journal.pcbi.1002628)
Supplement: Text S4 — Complete source code for the PyDSTool package (version 0.88.120504). Includes API documentation and help files linking to web pages. This file is identical to the current public release on Sourceforge.net. (ZIP) [file pcbi.1002628.s004.zip › PyDSTool/html/PyDSTool.Toolbox.phaseplane.mesh_patch_2D-class.html]

xml version="1.0" encoding="ascii"?


PyDSTool.Toolbox.phaseplane.mesh\_patch\_2D


| Home | Trees | Indices | Help | PyDSTool |
| --- | --- | --- | --- | --- |

Package PyDSTool ::
Package Toolbox ::
Module phaseplane ::
Class mesh\_patch\_2D

[hide private]

[frames] | no frames]

# Class mesh\_patch\_2D

source code

```
object --+
         |
        mesh_patch_2D
```

---

2D mesh patch generator (for 4 or 8 points of a fixed distance from a
central point). i.e., total number of mesh points may be 5 or 9.

Once either 5 or 9 points have been selected, this is fixed for the
mesh patch instance. The central point p0 and radii r of the mesh points
are mutable using the 'update' method. If shape='circle' (default), mesh
points are equidistant from p0. If shape='square', mesh points are
distributed around the corners and mid-sides of a square of side length
2\*r.

The points are in the order p0 followed by points in 'clockwise' order
in the phase plane defined by the two variable names in p0.

Functions / callable objects can be applied to all points in the mesh
patch by calling the mesh patch's 'eval' method with the function. It
returns an a dictionary of mesh points -> function return values.

The direction of minimum (interpolated) gradient of a functional over
the mesh can be determined from the 'min\_gradient\_dir' method. A similar
method 'max\_gradient\_dir' also exists.

Highly experimental class!

Rob Clewley, Jan 2007


|  |  |
| --- | --- |
| Instance Methods | [hide private] |

|  |  |
| --- | --- |
| \_\_init\_\_(self, p0, r, n=5, shape=`'``circle``'`)  p0 should be of type Point, r must be either a scalar or a Point giving weighted radius in each of two directions, n must be 5 or 9. | source code |

|  |  |
| --- | --- |
| make\_mesh(self, makelocalmesh=False) | source code |

|  |  |
| --- | --- |
| update(self, p0=None, r=None)  Change the central point p0 or patch radius r | source code |

|  |  |
| --- | --- |
| derive\_angles(self) | source code |

|  |  |
| --- | --- |
| \_\_getitem\_\_(self, ix) | source code |

|  |  |
| --- | --- |
| eval(self, f)  Evaluate the function or callable object f at the mesh patch points. | source code |

|  |  |
| --- | --- |
| setup\_grad(self, valdict, orient, dir) | source code |

|  |  |
| --- | --- |
| min\_gradient\_dir(self, valdict, orient=None, dir=None)  Find the direction of any local minima of a functional over the mesh patch, whose dictionary of mesh point index -> function value is given by valdict. | source code |

|  |  |
| --- | --- |
| min\_gradient\_mesh(self, valdict, orient=None, dir=None)  Find the direction of any local minima of a functional over the mesh patch, whose dictionary of mesh point index -> function value is given by valdict. | source code |

|  |  |
| --- | --- |
| \_\_min\_grad(self, valdict, orient, dir, return\_type) | source code |

|  |  |
| --- | --- |
| max\_gradient\_dir(self, valdict, orient=None, dir=None)  Find the direction of maximum gradient of a functional over the mesh patch, whose dictionary of mesh point index -> function value is given by valdict. | source code |

|  |  |
| --- | --- |
| max\_gradient\_mesh(self, valdict, orient=None, dir=None)  Find the direction of maximum gradient of a functional over the mesh patch, whose dictionary of mesh point index -> function value is given by valdict. | source code |

|  |  |
| --- | --- |
| \_\_max\_grad(self, valdict, orient, dir, return\_type) | source code |

|  |  |
| --- | --- |
| \_\_repr\_\_(self)  str(x) | source code |

|  |  |
| --- | --- |
| \_\_str\_\_(self)  str(x) | source code |

**Inherited from `object`**:
`__delattr__`,
`__getattribute__`,
`__hash__`,
`__new__`,
`__reduce__`,
`__reduce_ex__`,
`__setattr__`


|  |  |
| --- | --- |
| Class Variables | [hide private] |

\_s2 = `0.70710678118654757`

\_mesh5 = `[(0, 0), (0, 1), (1, 0), (0, -1), (-1, 0)]`

\_mesh9s = `[(0, 0), (0, 1), (1, 1), (1, 0), (1, -1), (0, -1), (...`

\_mesh9c = `[(0, 0), (0, 1), (0.70710678118654757, 0.70710678118...`


|  |  |
| --- | --- |
| Properties | [hide private] |

**Inherited from `object`**:
`__class__`


|  |  |
| --- | --- |
| Method Details | [hide private] |

|  |  |
| --- | --- |
| \_\_init\_\_(self, p0, r, n=5, shape=`'``circle``'`)  *(Constructor)* | source code |

p0 should be of type Point, r must be either a scalar or a Point
giving weighted radius in each of two directions, n must be 5 or 9.

Overrides: object.\_\_init\_\_

|  |  |
| --- | --- |
| eval(self, f) | source code |

```
Evaluate the function or callable object f at the mesh patch points.
Returns a dictionary of
    mesh point index -> function value
Indices are always in the same order as the Pointset attribute 'mesh'
```

|  |  |
| --- | --- |
| min\_gradient\_dir(self, valdict, orient=None, dir=None) | source code |

```
Find the direction of any local minima of a functional over
the mesh patch, whose dictionary of
    mesh point index -> function value
is given by valdict. The magnitudes of these values are
compared. The direction is indicated by a Point object, a
relative position at the patch's characteristic radius from
p0.

If the functional is constant everywhere on the patch (degenerate)
then a random direction is returned. If the functional is constant
everywhere except at the centre, then the vector (0,0) is returned
if the centre has lower value; a random vector is returned
otherwise.

If the functional is partially degenerate on some subset of
mesh points then the optional orient and dir arguments can
provide a means to select, provided the mesh resolution is
sufficiently high.

orient is a vector (Point object) relative to p0 orienting a
"positive" direction, such that the integer dir = -1 or +1
selects one half of the mesh points on which to conduct the
search.
```

|  |  |
| --- | --- |
| min\_gradient\_mesh(self, valdict, orient=None, dir=None) | source code |

```
Find the direction of any local minima of a functional over
the mesh patch, whose dictionary of
    mesh point index -> function value
is given by valdict. The magnitudes of these values are
compared. The direction is returned as a pair containing the two
closest mesh points to the direction (in mesh local coords).

If the functional is constant everywhere on the patch (degenerate)
then a random direction is returned. If the functional is constant
everywhere except at the centre, then the vector (0,0) is returned
if the centre has lower value; a random vector is returned
otherwise.

If the functional is partially degenerate on some subset of
mesh points then the optional orient and dir arguments can
provide a means to select, provided the mesh resolution is
sufficiently high.

orient is a vector (Point object) relative to p0 orienting a
"positive" direction, such that the integer dir = -1 or +1
selects one half of the mesh points on which to conduct the
search.
```

|  |  |
| --- | --- |
| max\_gradient\_dir(self, valdict, orient=None, dir=None) | source code |

```
Find the direction of maximum gradient of a functional over
the mesh patch, whose dictionary of
    mesh point index -> function value
is given by valdict. The magnitudes of the values are
compared. The direction is indicated by a Point object, a
relative position at the patch's characteristic radius from
p0.

If the functional is constant everywhere on the patch (degenerate)
then a random direction is returned. If the functional is constant
everywhere except at the centre, then the vector (0,0) is returned
if the centre has higher value; a random vector is returned
otherwise.

If the functional is partially degenerate on some subset of
mesh points then the optional orient and dir arguments can
provide a means to select, provided the mesh resolution is
sufficiently high.

orient is a vector (Point object) relative to p0 orienting a
"positive" direction, such that the integer dir = -1 or +1
selects one half of the mesh points on which to conduct the
search.
```

|  |  |
| --- | --- |
| max\_gradient\_mesh(self, valdict, orient=None, dir=None) | source code |

```
Find the direction of maximum gradient of a functional over
the mesh patch, whose dictionary of
    mesh point index -> function value
is given by valdict. The magnitudes of the values are
compared. The direction is returned as a pair containing the two
closest mesh points to the direction (in mesh local coords).

If the functional is constant everywhere on the patch (degenerate)
then a random direction is returned. If the functional is constant
everywhere except at the centre, then the vector (0,0) is returned
if the centre has higher value; a random vector is returned
otherwise.

If the functional is partially degenerate on some subset of
mesh points then the optional orient and dir arguments can
provide a means to select, provided the mesh resolution is
sufficiently high.

orient is a vector (Point object) relative to p0 orienting a
"positive" direction, such that the integer dir = -1 or +1
selects one half of the mesh points on which to conduct the
search.
```

|  |  |
| --- | --- |
| \_\_repr\_\_(self)  *(Representation operator)* | source code |

str(x)

Overrides: object.\_\_repr\_\_: *(inherited documentation)*

|  |  |
| --- | --- |
| \_\_str\_\_(self)  *(Informal representation operator)* | source code |

str(x)

Overrides: object.\_\_str\_\_: *(inherited documentation)*

  


|  |  |
| --- | --- |
| Class Variable Details | [hide private] |

### \_mesh9s

Value:
:   ```
    [(0, 0),
     (0, 1),
     (1, 1),
     (1, 0),
     (1, -1),
     (0, -1),
     (-1, -1),
     (-1, 0),
    ...
    ```

### \_mesh9c

Value:
:   ```
    [(0, 0),
     (0, 1),
     (0.70710678118654757, 0.70710678118654757),
     (1, 0),
     (0.70710678118654757, -0.70710678118654757),
     (0, -1),
     (-0.70710678118654757, -0.70710678118654757),
     (-1, 0),
    ...
    ```

  


| Home | Trees | Indices | Help | PyDSTool |
| --- | --- | --- | --- | --- |

|  |  |
| --- | --- |
| Generated by Epydoc 3.0.1 on Fri May 4 15:24:09 2012 | http://epydoc.sourceforge.net |
